# The Diagnostic Value of the Systemic Immune-Inflammation Index for Venous Thromboembolism in Lung Cancer Patients: A Retrospective Study

**DOI:** 10.1155/2022/9215311

**Published:** 2022-08-22

**Authors:** Lu Zhang, Xiangliang Liu, Ruihan Yang, Yi Yang, Xiao Chen

**Affiliations:** ^1^Department of Oncology, The First Affiliated Hospital of Anhui Medical University, Hefei, 230022 Anhui, China; ^2^Cancer Center, The First Hospital of Jilin University, Changchun, Jilin, China

## Abstract

**Background:**

Venous thromboembolism (VTE) is considered a common complication in lung cancer patients. Despite its widespread use, the Khorana score performed moderately in predicting VTE risk. This study aimed to determine the diagnostic utility of the Systemic Immunoinflammatory Index (SII) and to create a novel nomogram for predicting VTE in patients with pulmonary carcinoma.

**Materials and Methods:**

The data, like clinical features and laboratory indicators, of inpatients diagnosed with lung cancer from March 2019 to March 2020 were collected and analyzed. Univariate and multivariate logistic analyses were performed to confirm the risk factors and then construct a nomogram model. The calibration curve and clinical decision curve analysis (DCA) were used to assess the model's fitting performance. The receiver-operating characteristic (ROC) curve and the area under the ROC curve (AUC) were used to evaluate the diagnostic value of SII and the nomogram.

**Results:**

This study enrolled 369 lung patients with a VTE morbidity rate of 23.33%. The patients with VTE had higher SII levels than the non-VTE group (1441.47 ± 146.28 vs. 626.76 ± 26.04, *P* < 0.001). SII is the stronger correlator for VTE among inflammatory markers, of which the optimal cut-off value was 851.51. Univariate and multivariate analysis revealed that the age, metastasis, antitumor treatment, hemoglobin<100 g/L, SII>851.51 × 10^9^/L, and D-dimer>2 folds were independent risk factors for lung cancer-related VTE, and a new prediction nomogram model was constructed based on them. ROC curve analysis showed the AUC of the new model and Khorana score were 0.708 (0.643-0.772) and 0.600 (0.531-0.699).

**Conclusion:**

The SII was a simple and valuable biomarker for VTE, and the new nomogram model based on it can accurately forecast the occurrence of VTE. They can be utilized in clinical practice to identify those at high risk of VTE in lung cancer patients.

## 1. Introduction

Venous thromboembolism (VTE) is considered a secondary complication and the main cause of mortality in cancer patients, which consists of two major forms, deep venous thrombosis (DVT) and pulmonary embolism (PE) [1]. Patients with lung cancer are confronted with a substantially higher prevalence of VTE than those with other malignant solid tumors, in which the VTE rate ranges from 12% to 16.4% [2]. These patients should be evaluated for the likelihood of developing VTE as early as feasible to prevent their disease from being extremely convoluted, difficult to manage, or even fatal [3]. As a preventable disease, the cornerstone of clinical management of VTE is the early identification of high-risk groups. The Khorana score, the most canonical risk forecast model advocated by current guidelines, was widely used for VTE in patients with malignant tumors [4, 5], with the following items: diagnosed as pulmonary carcinoma, body mass index (BMI) ≥35 kg/m^2^, hemoglobin (HB) <100 g/L, white blood cells (WBC) >11 × 10^9^/L, and platelets ≥350 × 10^9^/L [6]. However, its clinical prediction accuracy has been contentious, so novel predictive markers specifically for lung cancer patients are required to be explored like never before [7].

Several studies have supported the tight relationship between immunity, inflammation, and thrombosis. The hyperinflammatory state in cancer patients' bodies generated enormous cytokines and inflammation mediators while stimulating the clotting response and ultimately promoting thrombosis [8]. Hematological parameters in peripheral blood, like lymphocyte ratio (NLR) [9], platelet to lymphocyte ratio (PLR) [10], and lymphocyte to monocyte ratio (LMR) [11], can render the host immune-inflammatory status and have been reported to correlate strongly with thrombotic events and the poor outcome of patients with cancer. However, certain studies illustrated that those biomarkers' sensitivity and efficacy were still in challenge [12]. The SII is a stable and widely available serological indicator, which integrates the counts of lymphocytes, neutrophils, and platelets [13]. It can offer a more balanced picture of the immunoinflammatory status and coagulation system [14].

Recently, some researchers have revealed that SII was a significant independent risk factor for PE [15], cerebral venous sinus thrombosis (CVT) [16], and portal vein thrombosis (PVT) [17], which can forecast the occurrence of venous thrombosis. However, the potential value of SII in predicting VTE risk in patients with lung cancer remains unclear. This study was designed to analyze the relevance of the inflammatory index and thrombosis events, evaluate its diagnostic value for lung cancer-related VTE, and finally to construct a nomogram prediction model to assist clinicians in accurately identifying people at high VTE risk in lung cancer patients.

## 2. Materials and Methods

### 2.1. Study Population

We retrospectively reviewed the data of patients hospitalized at the First Hospital of Jilin University from March 2019 to March 2020 through the electronic medical record system. Inclusion criteria were as follows: (1) patients with a pathology-confirmed diagnosis of primary pulmonary carcinoma; (2) patients who underwent at least one vessel ultrasound examination or computed tomography pulmonary angiography (CTPA) within 6 months of their diagnosis; (3) patients with comprehensive laboratory data, of which the peripheral blood was drawn before antineoplastic therapy and within three weeks after diagnosis; and (4) patients without severe impairment of bone marrow hematopoietic function, hepatic or renal function. Exclusion criteria were as follows: (1) patients with second primary carcinoma; (2) patients with severe infection; (3) patients with diseases that may potentially affect the peripheral blood cells; (4) patients with incomplete clinical records. This study was approved by the medical ethics committee of the First Hospital of Jilin University (No 2017-362) and all research processes adhered to the Helsinki Declaration. Given the retrospective nature of the research, the patients' consent was waived by the ethics committee.

### 2.2. Data Collection

Demographic data (gender, age, height, weight, etc.) and clinical information (pathological type, molecular subtype, staging, VTE events, etc.) were collected from medical records. The molecular subtypes were verified mostly in patients with lung adenocarcinoma, among which the epidermal growth factor receptor (EGFR), Kirsten rat sarcoma viral oncogene homolog (KRAS), c-ROS oncogene 1 (ROS1), and anaplastic lymphoma kinase (ALK) were confirmed by next-generation sequencing (NGS) tests. Peripheral blood laboratory like WBC, neutrophil (N), lymphocyte (L), monocyte (M), platelet count (PLT), HB, C-reactive protein (CRP), albumin (ALB), and D-dimer was gathered. The calculation formulas of some hematological indicators were as follows: NLR = N/L; PLR = PLT/L; LMR = L/M; SII=PLT×N/L.

### 2.3. Diagnosis of VTE

This study defined VTE as DVT, superficial thrombophlebitis, and PE. The DVT was diagnosed primarily through ultrasound imaging of thrombus formation in the lumen, hypoechoic regions, and disruption of blood flow signals. Superficial phlebitis is diagnosed mostly based on clinical symptoms, like redness, swelling, and pain exhibiting in the skin along the vessel's path, given no thrombotic masses in the vessel. The diagnosis of pulmonary embolism is based on the characteristic clinical symptoms as well as the filling defect of the CTPA. The above diagnosis was independently identified and reviewed by two experienced radiologists.

### 2.4. Statistical Analysis

All statistical analysis was conducted using IBM SPSS Statistics (22.0) and R software (4.1.3). Continuous variables with normal distribution were presented as mean ± standard deviation, which was compared by an independent Student's *t*-test. Continuous variables with nonnormal distribution were expressed as medians with interquartile range and compared by the Mann–Whitney test. Categorical variables were represented by cases and proportion, compared by chi-square or Fisher's exact test. Multivariate logistic regression analysis was performed for variables that had statistical significance in the univariate analysis. A nomogram model was constructed based on multivariate analysis through the “R” package. The receiver-operating characteristic (ROC) curve, calibration curve, and clinical decision curve analysis (DCA) were conducted using the “proc,” “resource selection,” and “rmda” packages. *P*-value<0.05 indicated that the difference was statistically significant.

## 3. Results

### 3.1. Patient Characteristics

A total of 369 patients with lung cancer were enrolled in this study with an average age of 60.05 (±9.24), including 181 males and 188 females. There were 86 cases diagnosed with VTE and 283 patients without VTE. The cumulative incidence of VTE within six months after lung cancer diagnosis was 23.33%. The clinical features of the two groups are shown in Table 1.

### 3.2. Hematological Parameters

**Figure 1:**
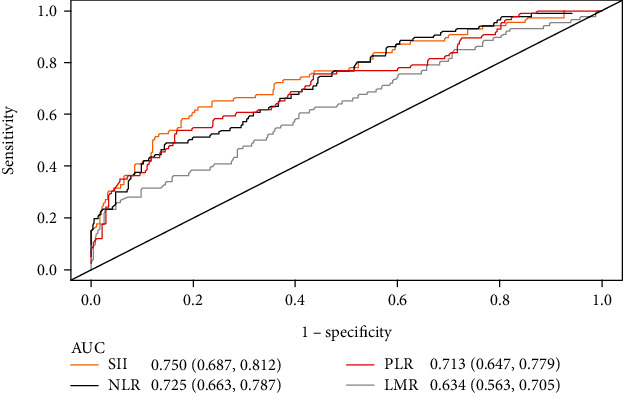
The receiver-operating characteristic (ROC) curve and the area under the ROC curve (AUC) of the systemic immune-inflammatory index (SII), neutrophil to lymphocyte ratio (NLR), platelet to lymphocyte ratio (PLR), and lymphocyte to monocyte ratio (LMR). The cut-off value of them was 851.51, 3.99, 198.86, and 2.20, respectively.

As shown in Table 2, the levels of hematologic parameters including WBC, N, L, PLT, NLR, PLR, SII, and D-dimer were higher (*P* < 0.005) in the VTE group, while HB was lower (*P* < 0.005) than those in the non-VTE group. The level of SII was higher in the VTE group than that of the non-VTE group (1441.47 ± 146.28 vs. 626.76 ± 26.04, *P* < 0.001). Furthermore, the analysis showed that the SII levels of patients with VTE at different phases varied in Table 3. In the acute phase, subacute phase, and chronic phase of VTE, the levels of SII were 1863.85 ± 246.31, 1209.11 ± 124.27, and 575.64 ± 58.38, respectively (*P* < 0.005). As shown in Figure 1, the AUC of SII, NLR, PLR, and LMR was 0.750 (0.687, 0.812), 0.725 (0.663, 0.787), 0.713 (0.647, 0.779), and 0.634 (0.563, 0.751). Their cut-off values were 851.51, 3.99, 198.86, and 2.20, respectively.

### 3.3. Univariate and Multivariate Analysis for Risk Factors

Continuous data were converted into categorical variables for analysis in consideration of published papers and articles on risk factors and the model establishment of VTE. Blood counts were labeled according to the Khorana score, D-dimers were grouped according to the degree of elevation, and other hematological data were classed using cut-off values of the ROC curve. BMI was defined based on the crucial value of obesity in Chinese individuals.

Firstly, the univariate logistic analysis showed that age, adenocarcinoma, gene mutation, metastasis, antitumor therapy, WBC, HB, PLT, CRP, NLR, PLR, LMR, SII, hypoproteinemia (Albumin <40 g/L), and D-dimer were correlated with VTE in lung cancer patients. These 15 variables with *P*-values less than 0.05 were included in the multifactorial analysis and the results are shown in Table 4. The result demonstrated that age >65 (OR =2.403, 95% CI: 1.321-4.370, *P* = 0.004), metastasis (OR =2.380, 95% CI: 1.329-4.261, *P* = 0.004), antitumor treatment (OR =2.414, 95% CI: 1.175-4.956, *P* = 0.016), HB <100 g/L (OR =3.844, 95% CI: 1.420-10.409, *P* = 0.008), SII (OR =3.355, 95% CI: 1.849-6.088, *P* < 0.001), and D-dimer (OR =4.083, 95% CI: 2.238-7.447, *P* < 0.001) were independent prediction factors of VTE in patients with lung cancer. It highlighted that patients with SII >851.51 × 10^9^/L had a 3.36-fold higher hazard of incurring VTE than those with SII ≤851.51 × 10^9^/L.

### 3.4. Development and Verification of Nomogram Model

**Figure 2:**
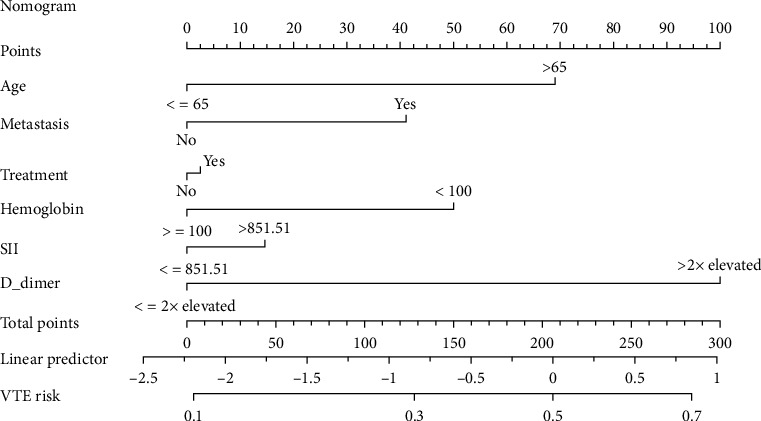
The nomogram for predicting VTE risk in lung cancer patients based on the systemic immune-inflammatory index (SII). Different scores were obtained for each variable, after which the total scores of the 6 items were assessed for the corresponding VTE predicted risk.

**Figure 3:**
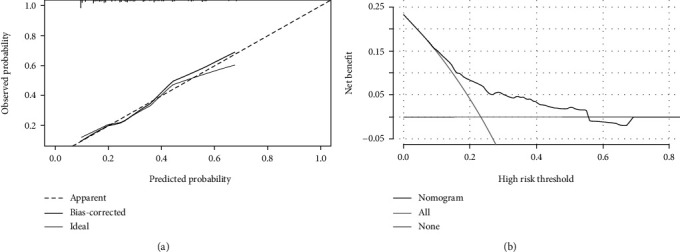
Evaluation of the nomogram. (a) Calibration plot of the nomogram for VTE in lung cancer (bootstrap 1000 repetitions). (b) Decision curve analysis (DCA) of the nomogram for VTE in lung cancer.

**Figure 4:**
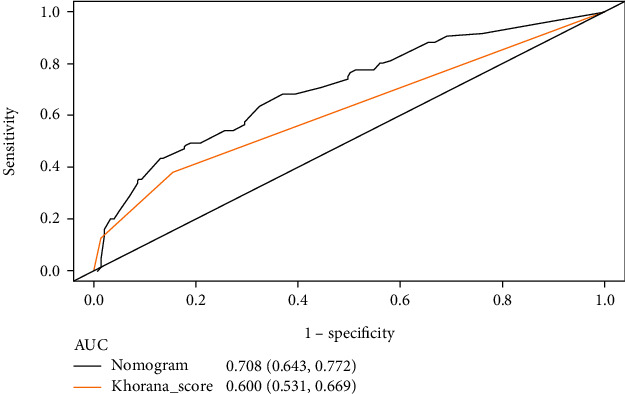
The receiver-operating characteristic (ROC) curve and the area under the ROC curve (AUC) of the nomogram and the Khorana score. Their AUCs were 0.708 and 0.600.

This study established a nomogram prediction model for VTE in lung cancer patients based on the results of the multivariate analysis as shown in Figure 2. Different scores were obtained for each variable, after which the total scores of the 6 items were assessed for the corresponding VTE predicted risk. The calibration plot in Figure 3(a) and the Hosmer-Lemeshow goodness-of-fit test indicated that the model's calibration is reasonable (*P* = 0.427). The DCA curve in Figure 3(b) illustrated that the nomogram model had an excellent clinical application. The internal verification for the nomogram through the bootstrap method with 1000 repetitions of sampling and the AUC was 0.708 (0.643, 0.772), while the AUC Khorana score was 0.600 (0.531, 0.669) as shown in Figure 4. This demonstrated that the new nomogram model had excellent prediction performance.

## 4. Discussion

Venous thromboembolism is an extremely widespread and possibly lethal illness. It is a common complication and the leading cause of non-neoplastic death in patients with malignant tumors, accounting for more than 3 million fatalities each year. Patients with lung cancer are exposed to a higher risk of VTE, of which the prevalence ranges from 12% to 16.4% [3]. It is necessary to explore simple and reliable biomarkers to determine the VTE risk in lung cancer individuals.

Inflammatory indicators were considerably increased in VTE patients than in non-VTE patients in our study, both in terms of peripheral blood count and NLR, PLR, LMR, and SII, indicating a close bond between inflammatory status and thrombosis. The ROC curve and logistic regression analysis demonstrated that SII was the exclusive independent risk variable with the maximum AUC. The optimal cut-off value of SII was 851.51, which meant patients with SII >851.51 face a higher risk of developing VTE. Based on the finding that SII was the stronger correlator for VTE, we developed a nomogram model and internally validated its excellent diagnostic efficacy. Several recent studies have supported our conclusion that SII provided clinical diagnostic value for VTE. Gok et al. observed a total of 442 cases with acute pulmonary embolism (APE), of which SII was an independent predictor (OR =1.005, 95% CI: 1.002-1.007, *P* < 0.001) [15]. They found that SII was significantly higher in APE patients, and the degree of enrichment of SII was positively correlated with the severity of APE [15]. Another two single-center retrospective studies determined a linkage between SII and thrombosis. The area under AUC was 0.827 and the cut-off point was 496.07 in CVT patients [16], while AUC was 0.612 and the cut-off value was 268.9 in PVT cases [17]. Peng et al. probed SII as an available predictor for VTE after hip fracture in the elderly (OR =1.004, 95% CI: 1.001–1.008, *P* = 0.001), of which the AUC was 0.795 and the cut-off point was 847.78 [18]. Posterior two studies simultaneously manifest that the model based on SII had good prediction performance. Furthermore, we discovered that SII level appeared to be higher in the acute phase of thrombosis than in the subacute or chronic phase by comparing with SII in several researches [15], which was consistent with our study. This finding suggested that individuals in the acute phase may have the intense systemic inflammatory response in vivo. This may be the result of a systemic inflammatory hyper-reactive state promoting thrombosis, as well as local thrombosis in the vessels promoting the release of inflammatory factors.

Multiple clinical studies demonstrated that SII did have a strong predictive value for VTE, while fundamental research provided theoretical support for the association of the inflammatory response with thrombosis [19]. Altered systemic inflammatory responses and active coagulation systems were encountered in patients with lung cancer, which were closely related to the development and progression of VTE [20]. Circulating neutrophils, lymphocytes, and platelets were considered systemic inflammatory immune cells of the human body, which not only stimulated tissue factors that trigger coagulation by secreting a vast group of inflammatory mediators like interleukin (IL)-1*β* and IL-6 but also enhanced the activation of nod-like receptor protein three inflammatory vesicles (NLRP3) inflammatory vesicles to promote thrombus formation [21, 22]. Moreover, it is reported that inflammatory cytokines and adhesion molecules as inflammatory mechanisms contributed to endothelial injury and dysfunction, in parallel with neutrophil extracellular trap formation, endothelial cell, and monocyte activation [23, 24]. Ultimately, activation of coagulation pathways put the body into a pro-thrombotic state [25]. Recent work has implicated local vascular thrombo-inflammation may be another vital pathogenetic mechanism of VTE in cancer patients [26]. SII was recently acknowledged as a stronger predictor of clinical prognosis for multiple malignancies, with a comprehensive combination of neutrophils, lymphocytes, and platelets [27–29].

The Khorana score performed poorly in predicting VTE, particularly in lung cancer patients who may be at high risk of VTE [30]. To tackle this challenge, the prediction nomogram was generated based on the diagnostic value of SII for lung cancer-associated VTE and combining patient clinical characteristics (e.g., age, metastasis, antitumor treatment, and D-dimer). Preliminary tests illustrated that the model with rational variables and straightforward operations had outstanding prediction performance. Several studies have identified age as an independent risk factor for venous thrombotic events [31]. The VTE risk in advanced cancer patients with metastases, signaling the substantial tumor burdens, was three times higher than those in non-metastatic patients [32]. Antitumor drugs caused the body to become hypercoagulable, whereas therapeutic approaches induced vascular endothelial damage. Alterations in hemodynamics, damage to the vessel walls, and hypercoagulability were all significant determinants of intravascular thrombosis, which was also known as Virchow's triad [33]. Aside from the extremely inflammatory responses that accelerated endothelial injury, most lung cancer patients presented with hypercoagulable states making them a high risk for VTE [34]. The D-dimer was an excellent biomarker for sensitively detecting alterations in the coagulation system and was widely applied in clinical practice to exclude or identify thrombosis [35].

However, there are some existing limitations in our current study that impede the interpretation of the findings. First and foremost, this was a single-center retrospective clinical study presented with a small sample volume of which merely 369 patients in total and only 86 cases with VTE. Secondly, there was a shortage of sufficient external validation targeting the new predictive model. In addition, we did not probe the prognosis value of the enrolled subjects on account of the low follow-up rate. Considering the above limitations, some prospective clinical trials with sufficient samples need to be designed in the future to evaluate the diagnostic and prognostic value of SII for lung cancer-related VTE.

## 5. Conclusion

SII was a straightforward and valuable predictor for VTE events in lung cancer patients, especially for acute or subacute VTE. The new nomogram model, which consisted of the inflammatory marker, coagulation indicator, and tumor features, delivered an intuitive and accurate prediction of VTE. It could be used in clinical practice to identify lung cancer patients at high risk of VTE.

## Figures and Tables

**Table 1:** Comparison of clinical data between VTE groups and non-VTE groups.

Variable	VTE (*N* = 86)	Non-VTE group (*N* = 283)	*χ*2	*P*-value
Gender			1.631	0.202
Female	37 (57.0%)	144 (50.9%)		
Male	49 (43.0%)	139 (49.1%)		
Age			11.929	<0.001
>65	39 (45.3%)	73 (25.8%)		
≤65	47 (54.7%)	210 (74.2%)		
BMI			0.407	0.524
>28	6 (7.0%)	26 (9.2%)		
≤28	80 (93.0%)	257 (90.8%)		
Smoking			0.206	0.650
Yes	35 (40.7%)	123 (43.5%)		
No	51 (59.3%)	160 (56.5%)		
Vessel disease			0.006	0.936
Yes	1 (1.2%)	3 (1.1%)		
No	85 (98.8%)	280 (98.9%)		
Pathological type			6.608	0.037
Adenocarcinoma	53 (61.6%)	133 (47.0%)		
SCC	21 (24.4%)	80 (28.3%)		
SCLC	12 (14.0%)	70 (24.7%)		
Metastasis			19.47	<0.001
Yes	47 (55.3%)	82 (29.2%)		
No	38 (44.7%)	199 (70.8%)		
Molecular subtypes			9.574	0.002
Wild	57 (66.3%)	232 (82.0%)		
Mutation	29 (33.7%)	51 (18.0%)		
Antitumor therapy			5.611	0.018
Yes	70 (81.4%)	193 (68.2%)		
No	16 (18.6%)	90 (31.8%)		

Notes: Abbreviations: ALK: the anaplastic lymphoma kinase; EGFR: the epidermal growth factor receptor; KRAS: the Kirsten rat sarcoma viral oncogene homolog; ROS1: the c-ROS oncogene 1; SCC: squamous cell carcinoma; SCLC: small cell lung cancer.

**Table 2:** Comparison of laboratory results between VTE groups and non-VTE groups.

Variable	VTE (*N* = 86)	Non-VTE group (*N* = 283)	*T*/*Z*	*P*-value
WBC (×10^9^/L)	7.52 (5.46, 9.80)	5.90 (4.80, 7.57)	-5.396	<0.001
N (×10^9^/L)	5.05 (3.92, 7.48)	3.71 (2.91, 5.11)	-4.447	<0.001
L (×10^9^/L)	1.37 (1.02, 1.74)	1.58 (1.24, 2.01)	-3.031	0.002
M (×10^9^/L)	0.44 (0.28, 0.64)	0.39 (0.28, 0.52)	-1.900	0.057
HB (g/L)	126 (±22.91)	138.08 (±19.09)	4.248	<0.001
PLT (×10^9^/L)	266.50 (209.75, 341.50)	216.00 (174.00, 281.00)	-4.211	<0.001
CRP (mg/L)	10.40 (3.23, 34.70)	4.28 (3.13, 19.90)	-2.675	0.007
Albumin (g/L)	35.89 (±5.54)	38.96 (±4.04)	4.752	<0.001
D-dimer (mg)	754 (375, 2126)	247.59 (139.75, 559.00)	-6.824	<0.001
NLR	3.54 (2.54, 5.60)	2.46 (1.69, 3.36)	-6.327	<0.001
PLR	200.49 (146.28, 297.56)	133.84 (102.83, 187.22)	-5.990	<0.001
LMR	3.35 (1.90, 4.60)	4.05 (3.03, 5.42)	-3.764	0.007
SII (×10^9^/L)	1090.34 (602.82, 1817.95)	530.31 (318.59, 770.01)	-7.018	<0.001

Notes: CRP: C-reactive protein; HB: hemoglobin; L: lymphocyte; LMR: lymphocyte to monocyte ratio; M: monocyte; N: neutrophil; NLR: neutrophil to lymphocyte ratio; PLR: platelet to lymphocyte ratio; PLT: platelet count; SII: systemic immune-inflammation index; WBC: white blood cell count.

**Table 3:** SII levels of patients with VTE at different phases.

Clinical stage of VTE	Case (%)	SII	*P*-value
Acute phase	46 (53.48%)	1863.85 (±246.31)	<0.001
Subacute phase	24 (27.90%)	1209.11 (±124.27)	
Chronic phase	16 (18.60%)	575.64 (±58.38)	

**Table 4:** Comparison of clinical data between VTE groups and non-VTE groups.

Variate	Univariate		Multivariate	
	OR (95% CI)	*P*-value	OR (95% CI)	*P*-value
Female	1.372 (0.844-2.231)	0.203		
Age>65	2.378 (1.446-3.940)	0.001	2.403 (1.321-4.370)	0.004
BMI>28	1.349 (0.536-3.393)	0.525		
Smoking	0.893 (0.547-1.458)	0.650		
Vessel disease	1.098 (0.113-10.694)	0.936		
Pathological type		0.040		
Adenocarcinoma	2.325 (1.166-4.635)	0.017		
SCC	1.531 (0.703-3.335)	0.283		
Metastasis	3.002 (1.822-4.944)	<0.001	2.380 (1.329-4.261)	0.004
Mutation	2.314 (1.349-3.972)	0.002		
Antitumor therapy	2.040 (1.122-3.710)	0.019	2.414 (1.175-4.956)	0.016
WBC>11 × 10^9^/L	5.920 (2.464-14.225)	<0.001		
HB<100(g/L)	3.422 (1.541-7.600)	0.003	3.844 (1.420-10.409)	0.008
PLT>350 × 10^9^/L	2.732 (1.409-5.297)	0.003		
CRP>7.29 mg/L	2.078 (1.274-3.387)	0.003		
Hypoproteinemia	2.326 (1.337-4.045)	0.003		
D-dimer>2 folds	5.856 (3.454-9.928)	<0.001	4.083(2.238-7.447)	<0.001
NLR>3.99	5.634 (3.293-9.639)	<0.001		
PLR>198.86	4.896 (2.898-8.272)	<0.001		
LMR>2.20	3.946 (2.159-7.215)	<0.001		
SII>851.51 × 10^9^/L	6.407 (3.798-10.808)	<0.001	3.355 (1.849-6.088)	<0.001

Notes: OR: the odds ratio.

## Data Availability

The data used to support the findings of this study are available from the corresponding author upon request.
